# Validation of multiplex immunofluorescence panels using multispectral microscopy for immune-profiling of formalin-fixed and paraffin-embedded human tumor tissues

**DOI:** 10.1038/s41598-017-13942-8

**Published:** 2017-10-17

**Authors:** Edwin R. Parra, Naohiro Uraoka, Mei Jiang, Pamela Cook, Don Gibbons, Marie-Andrée Forget, Chantale Bernatchez, Cara Haymaker, Ignacio I. Wistuba, Jaime Rodriguez-Canales

**Affiliations:** 1 Departments of Translational Molecular Pathology, The University of Texas MD Anderson Cancer Center, Houston, Texas USA; 2 Thoracic/Head and Neck Medical Oncology, The University of Texas MD Anderson Cancer Center, Houston, Texas USA; 3 Melanoma Medical Oncology, The University of Texas MD Anderson Cancer Center, Houston, Texas USA

## Abstract

Immune-profiling is becoming an important tool to identify predictive markers for the response to immunotherapy. Our goal was to validate multiplex immunofluorescence (mIF) panels to apply to formalin-fixed and paraffin-embedded tissues using a set of immune marker antibodies, with the Opal™ 7 color Kit (PerkinElmer) in the same tissue section. We validated and we described two panels aiming to characterize the expression of PD-L1, PD-1, and subsets of tumor associated immune cells. Panel 1 included pancytokeratin (AE1/AE3), PD-L1, CD4, CD8, CD3, CD68, and DAPI, and Panel 2 included pancytokeratin, PD-1, CD45RO, granzyme B, CD57, FOXP3, and DAPI. After all primary antibodies were tested in positive and negative controls by immunohistochemistry and uniplex IF, panels were developed and simultaneous marker expressions were quantified using the Vectra 3.0™ multispectral microscopy and image analysis InForm™ 2.2.1 software (PerkinElmer).These two mIF panels demonstrated specific co-localization in different cells that can identify the expression of PD-L1 in malignant cells and macrophages, and different T-cell subpopulations. This mIF methodology can be an invaluable tool for tumor tissue immune-profiling to allow multiple targets in the same tissue section and we provide that is accurate and reproducible method when is performed carefully under pathologist supervision.

## Introduction

Novel and effective immunotherapies for patients with various types of cancer are becoming a clinical reality, in part because of the remarkable clinical efficacy observed with immune checkpoint inhibitors such as programmed cell death protein 1 (PD-1, a T-cell co-inhibitory receptor) and one of this protein’s ligands, programmed cell death ligand 1 (PD-L1, also known as B7-H1 or CD274)^1–12^. These inhibitors are used to analyze the tumor microenvironment in patients with various types of cancer, a step fundamental to recognizing the details of the tumor-host interaction, leading to the development of therapies^1,13^. Characterization of the tumor microenvironment in patients with cancer has become a fundamental step in discovering evidence for the presence of distinct immunologic phenotypes, based on the presence or absence of various immune cells^1,13,14^. These observations have generated candidate predictive biomarkers that can respond to immunotherapies and are guiding the identification of new immunotherapeutic interventions^15^. Tumor-associated immune cells (TAICs) may respond to therapies targeting immune system inhibitory or stimulatory mechanisms, and non-TAICs may require additional interventions aimed at promoting optimal inflammation and innate immune activation in the tumor microenvironment^16–18^. Characterizing and validating these multiplex immunofluorescence (mIF) staining using immune system–based biomarkers has several critical implications for clinical translation and has emerged as a more potent tool for immunoprofiling analysis, offering simultaneous detection of multiple markers in the same tissue section in formalin-fixed and paraffin-embedded (FFPE) tumor tissues to deeper understanding the tumor microenvironment.

In the current study, our goal was to validate mIF panels in the same tissue section to apply to FFPE carcinoma tissues using a set of immune marker antibodies, including those against PD-L1 and TAICs, multispectral microscopy and image analysis software.

## Materials and Methods

### FFPE tissue specimens

Sequential 4-µm-thick sections from Hodgkin disease–derived cell line (HDLM-2/PD-L1 positive, SignalSlide #13747, Cell Signaling Technology, Danvers, MA), prostate cancer cell line (PC3/PD-L1 negative, SignalSlide #13747, Cell Signaling Technology), human mature placenta and human tonsil FFPE tissues were prepared for conventional immunohistochemistry (IHC), uniplex and multiplex IF validation. Additionally, sequential 4-µm-thick sections from cases of non–small cell lung carcinoma (NSCLC, 10 cases), adenocarcinoma (5), and squamous cell carcinoma (5) were prepared for conventional IHC and mIF staining.

### Immunohistochemistry validation

Chromogen-based IHC analysis was performed by using an automated staining system (BOND-MAX; Leica Microsystems, Vista, CA) with antibodies against the following: pancytokeratin AE1/AE3 (epithelial cell positive, dilution 1:300, Dako, Carpinteria, CA), PD-L1 (clone E1L3N, dilution 1:100; Cell Signaling Technology), CD4 (helper T cells, Novocastra, clone 4B12, dilution 1:80, Leica Biosystems, Buffalo Grove, IL; CD4 clone SP35, ready to use, Ventana Medical Systems, Tucson, AZ; CD4 clone SP35, dilution 1:100, Spring Bioscience, San Francisco, CA), CD8 (cytotoxic T cells, clone C8/144B, dilution 1:20; Thermo Fisher Scientific, Waltham, MA), CD3 (T-cell lymphocytes, dilution 1:100; Dako), CD68 (macrophages, clone PG-M1, dilution 1:450; Dako), PD-1 (clone EPR4877-2, dilution 1:250; Abcam, Cambridge, MA), granzyme B (cytotoxic lymphocytes, clone F1, ready to use; Leica Biosystems), CD57 (natural killer T cells, clone HNK-1, dilution 1:40; BD Biosciences, San Jose, CA), CD45RO (memory T cells, clone UCHL1, ready to use; Leica Biosystems), and FOXP3 (regulatory T cells, clone 206D, dilution 1:50; BioLegend, San Diego, CA). Expression of all cell markers was detected using a Novocastra Bond Polymer Refine Detection Kit (Leica Microsystems, catalogue #DS9800) with a diaminobenzidine reaction to detect antibody labeling and hematoxylin counterstaining. The correct titrations of antibodies in IHC analysis were chosen on the basis of the minimum to maximum range of staining negative to positive in the control specimens, combined with the uniformity of staining within the specific cell expression with the different antibodies to obtain a correct staining pattern. Positive and negative controls were used for PD-L1 IHC analysis validation: HDLM-2 cell line, human mature placenta and human tonsil as positive controls, and PC3 cell line as negative control. For the TAICs IHC expression, human tonsil FFPE tissues with and without primary antibody were used as positive and negative controls, in each batch IHC staining.

### IF validation of antibodies

After chromogen-based IHC analysis was used for all of the targets, each target was assessed by a uniplex IF assay to optimize the antibodies and to generate spectral libraries required for multiplex IF image analysis. Uniplex IF staining was performed manually by using the Opal 7 kit (catalogue #NEL797001KT; PerkinElmer, Waltham, MA), which uses individual tyramide signal amplification (TSA)-conjugated fluorophores to detect various targets within an IF assay. After deparaffinization, slides were placed in a plastic container filled with antigen retrieval (AR) buffer in Tris-EDTA buffer (for CD4, CD3, granzyme B, and CD57 analysis) or citrate buffer (for analysis of the remaining markers); microwave technology (EZ-RETRIEVER^®^ system microwave from BioGenex) was used to bring the liquid to the boiling point (1 min) at 100 °C, and the sections were then microwaved for an additional 15 min at 75 °C. Slides were allowed to cool in the AR buffer for 15 min at room temperature and were then rinsed with deionized water and 1 × Tris-buffered saline with Tween 20 (TBST; Santa Cruz Biotechnology, Dallas, TX). To initiate protein stabilization and background reduction, Tris-HCl buffer containing 0.1% Tween (Dako, catalogue #S3022) was used for 10 min at room temperature. Slides were then incubated between 30 min and 2 h (depending on which antibody was used at room temperature) with the same primary antibodies used for IHC analysis against the immune markers at specific dilutions: AE1/AE3 (dilution 1:300), PD-L1 (dilution 1:3000), CD4 (dilution 1:80), CD8 (dilution 1:120), CD3 (dilution 1:100), PD-1 (dilution 1:250), granzyme B (dilution 1:1), CD57 (dilution 1:10), CD45RO (dilution 1:1), FOXP3 (dilution 1:50), and CD68 (dilution 1:450). Next, the slides were washed and incubated for 10 min at room temperature with anti-mouse or anti-rabbit secondary antibodies (Novocastra, Leica Biosystems) after successive washes in TBST.

The slides were then incubated at room temperature for 10 min with one of the following Alexa Fluor tyramides (PerkinElmer) included in the Opal 7 kit to detect antibody staining, prepared according to the manufacturer’s instructions: Opal 520, Opal 540, Opal 570, Opal 620, Opal 650, and Opal 690 (dilution 1:50). After three additional washes in deionized water, the slides were counterstained with DAPI for 5 min and mounted with VECTASHIELD Hard Set (Vector Labs, Burlingame, CA). Autofluorescence (negative control) slides were also included, using primary and secondary antibodies and omitting the fluor tyramides. As performed with the IHC staining, the correct titration in the uniplex IF slides was chosen carefully to obtain a uniform, specific, and correct staining pattern. Similar to IHC validation, positive and negative controls were used during each run staining.

### Multiplex IF staining

Once each target was optimized in uniplex slides, the Opal 7 multiplexed assay was used to generate multiple staining slides. We applied primary antibodies to tonsil specimens as controls at optimized concentrations previously determined on the uniplex control tissues. Staining was performed consecutively by using the same steps as those used in uniplex IF, and the detection for each marker was completed before application of the next antibody. The best sequence of antibodies for multiplex staining was determined for each panel combination: panel 1 [pancytokeratin AE1/AE3, PD-L1, CD4 (clone 4B12), CD8, CD3, CD68, and DAPI] and panel 2 (pancytokeratin AE1/AE3, PD-1, CD45RO, granzyme B, CD57, FOXP3, and DAPI).

To analyze possible variations in staining over time, slide tissues from a set of 10 sequential FFPE tissues from NSCLC were stained at various 1-week intervals. Over 3 consecutive weeks, a series of 12 slides, including control (tonsil) and autofluorescence tissue (negative control), was stained by the same technician; these included panel 1 and panel 2, and slides were stained while maintaining the same conditions and using the same Opal 7 kit. At the same time, consecutive slide tissues were stained with chromogenic IHC stain in the autostainer with the use of the same markers with the dilutions mentioned previously.

### Image collection and analysis

Upon completion of the chromogenic IHC analysis from individual markers, uniplex and multiplex IF staining, the slides were imaged using the Vectra 3.0 spectral imaging system (PerkinElmer) according to previously published instructions^19^. The chromogenic IHC-stained slides were scanned by using the brightfield protocol, and the uniplex and multiplex IF staining was imaged by using the fluorescence protocol at 10 nm *λ* from 420 nm to 720 nm, to extract fluorescent intensity information from the images. A similar approach was used to build the spectral library using the InForm 2.2.1 image analysis software (PerkinElmer). In the multiplex IF slides, each batch was scanned with the Vectra imaging system using a tonsil as a control to calibrate the spectral image protocol. After low magnification scanning at × 10, the specimens were sampled from five individual fields (0.669 × 500 µm, 0.3345 mm^2^ each) randomly in the tonsil and in the intratumoral compartment by using the phenochart 1.0.4 (PerkinElmer) viewer to scan at high resolution ( × 20) in order to capture various elements of tonsil and tumor heterogeneity. Histologic assessment of each analysis area was performed to ensure that the tumor tissue (at least 85% malignant cells, AE1-/AE3-positive, and tumor stroma) was included in the selected intratumoral region. For this analysis, each area examined was overlapped with the sequential mIF and IHC slides to quantify each marker at the same location of the tonsil or tumor specimens.

The data from the multispectral camera were accessed by the imaging InForm software, and then each individual unmixed staining was combined by using the spectral library information to associate each fluorochrome component with a mIF component. All the immune cell populations from each panel were characterized and quantified using the cell segmentation and phenotype cell tool by the InForm image analysis software under pathologist supervision (ERP and NU). The individual markers from each mIF panels as well as from the chromogenic IHC was quantified and the average of the different number of PD-L1 and TAIC subpopulations from the five areas was expressed in density per mm^2^.

### Statistical analysis

The Spearman’s test was used to detect differences in continuous variables between the different staining batches and between mIF and chromogenic IHC quantifications. The statistical software program IBM SPSS (version 22; Armonk, NY) was used to perform the computations for all analyses.

## Results

### IHC and uniplex IF validation

**Figure 1:**
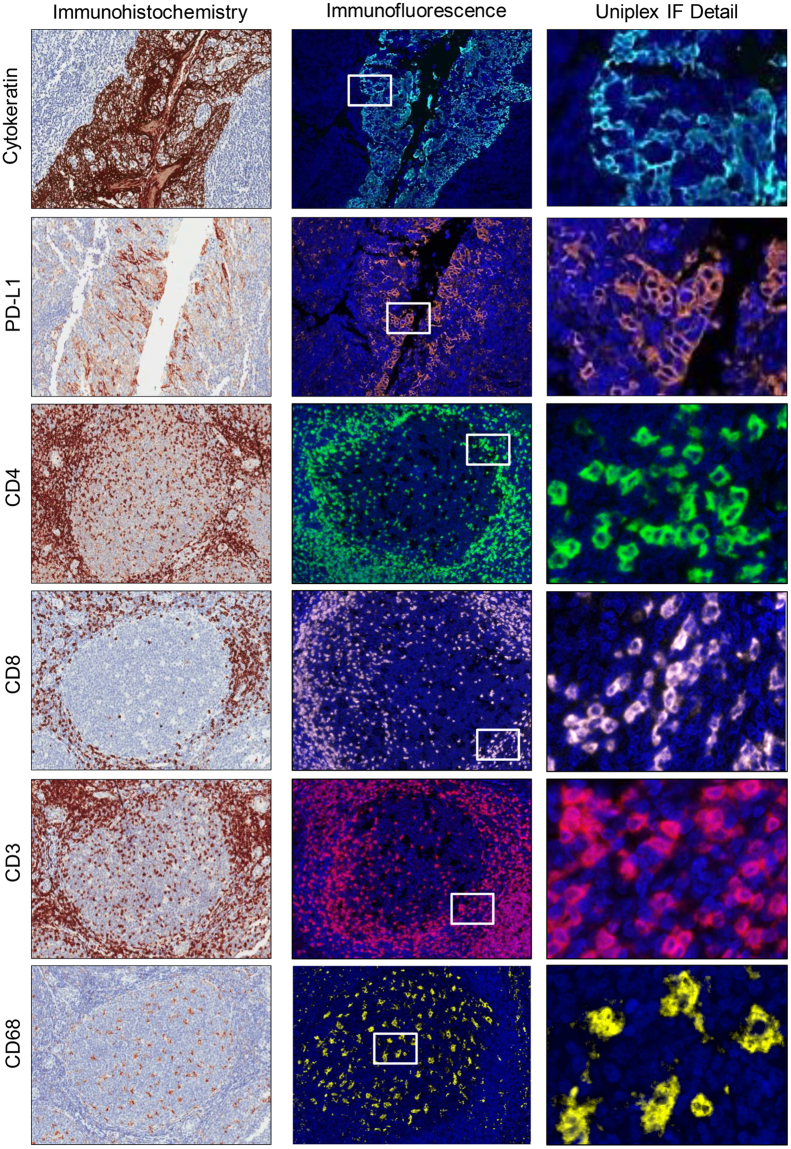
Microphotographs of representative examples of validation from IHC (*left panels*), uniplex IF tumor-associated immune cell expression (*middle panels*), and details of uniplex IF in tonsil tissue (*right panels*). Immune panel 1: AE1/AE3 (cytokeratin-positive), PD-L1–positive, helper T cell (CD4-positive), cytotoxic T cell (CD8-positive), T-cell lymphocyte (CD3-positive), and macrophages (CD68-positive). ×200 magnification and high-power magnification of the positive cells.

**Figure 2:**
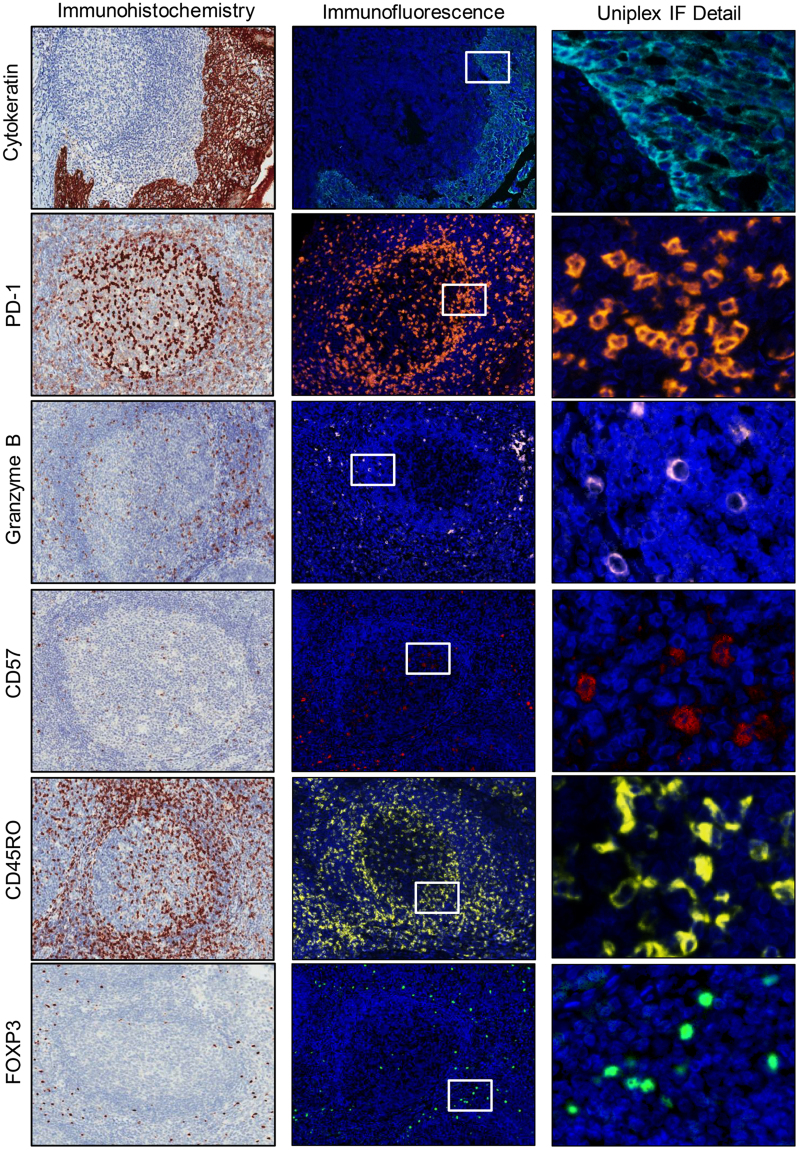
Microphotographs of representative examples of validation from uniplex IHC (*left panels*), uniplex IF tumor-associated immune cell expression (*middle panels*), and details of uniplex IF in tonsil tissue (*right panels*). Immune panel 2: AE1/AE3 (cytokeratin-positive), PD-1–positive, granzyme B–positive, natural killer cell (CD57-positive), memory T cell (CD45RO-positive), and regulatory T cell (FOXP3-positive). ×200 magnification and high-power magnification of the positive cells.

Using chromogenic IHC and uniplex IF approaches, we evaluated the different markers to obtain similar patterns of staining with both immunohistochemical techniques. As previously reported^20^, PD-L1 showed membranous expression in epithelial tonsil crypts, and in placenta syncytiotrophoblasts, as shown in the microphotographs in Supplementary Figure 1A and B, respectively. The specificity of PD-L1 expression was also tested with the use of the HDLM2 cell line as a positive control and the PC3 cell line as a negative control. Membrane PD-L1 expression was observed in HDLM2 cells, but no expression was observed in PC3 cells (Supplementary Figure 1C and D, respectively). Likewise, the other markers—pancytokeratin AE1/AE3 (epithelial cell–positive), helper T cells (CD4-positive), cytotoxic T cells (CD8-positive), T-cell lymphocytes (CD3-positive), granzyme B–positive, natural killer cells (CD57-positive), macrophages (CD68-positive), PD-1–positive, effector and/or memory T cells (CD45RO-positive), and regulatory T cells (FOXP3-positive)—showed similar staining patterns with IF uniplex staining compared with IHC stains in the tonsil controls (Figs 1 and 2).

### Multiplex IF Validation

**Figure 3:**
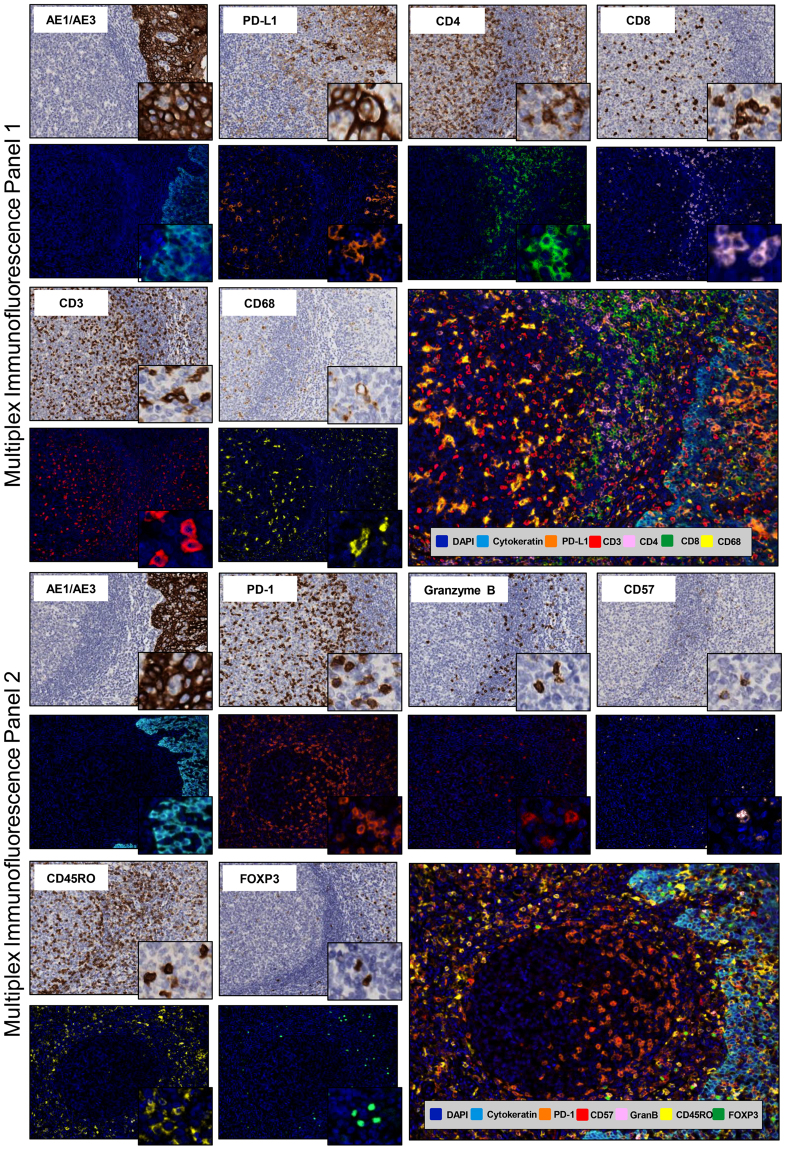
Microphotographs of representative examples of individual IHC and multiplex IF markers in tonsil tissue. Immune panels 1 and 2. ×200 magnification and high-power magnification of the positive cells.

After testing all of the markers by chromogenic IHC and uniplex IF staining, we validated the markers by multiplex IF staining in two separate panels. Tonsil was used as a positive control to explore the various markers in specific distributions. The first panel (pancytokeratin AE1/AE3, PD-L1, CD4, CD8, CD3, CD68, and DAPI) and the second panel (pancytokeratin AE1/AE3, PD-1, CD45RO, granzyme B, CD57, FOXP3, and DAPI) showed specific staining without background staining in the major part of the markers (Fig. 3). Unfortunately, all the CD4 marker clones tested in the mIF showed a diffuse granular membrane staining with different levels of background in the tissue. The other individual markers in each panel maintained a pattern distribution similar to that observed in the IHC tonsil (positive control) staining (Fig. 3).

### Application of multiplex IF to non-small cell carcinoma specimens

After testing the immune panels in control tissues and comparing them with chromogenic IHC patterns from each marker, we applied these panels in a set of NSCLC tissues. Ten NSCLC specimens were stained with the mIF panels in three different batches in consecutive weeks (Supplementary Figure 2) to observe possible variations in the staining and to compare the densities of immune cells analyzed in these specimens with the densities of those cells analyzed in the individual chromogenic IHC staining. Quantification of the various cell populations using mIF and chromogenic IHC is shown in Table 1. Overall, the correlation analysis between cell densities of each mIF batch and IHC stained batch showed significant positive correlations as shown in Supplementary Table 2 and Supplementary Figure 3. In batch 1 vs IHC, we observed positive and significant correlations between PD-L1, CD8, CD4, CD3, PD-1, and CD57 markers (r = 0.846 to r = 0.618, *P* < 0.05). In batch 2 vs IHC, we observed significant positive correlations between AE1/AE3, PD-L1, CD4, CD3, and PD-1 markers (r = 0.918 to r = 0.655, *P* < 0.05). In batch 3 vs IHC, we observed significant positive correlations between PD-L1, CD4, CD8, CD3, CD68, PD-1, and CD57 markers (r = 0.909 to r = 0.627, *P* < 0.05). The correlation analysis showed that the more reliable consistent markers in terms of density reproducibility between each mIF batch and chromogenic IHC included PD-L1, CD3, CD4, and PD-1, followed by CD8 and CD57, which showed a significant trend (r = 0.555, *P* = 0.077; r = 0.527, *P* = 0.096, respectively) in the second batch. Despite the background with the CD4 marker in the mIF, the correlations with the chromogenic IHC were positive and significant (Supplementary Table 2).

**Table 1:** Densities of immune markers (in mm^2^) in the various staining batches.

Marker	Median density ( ± SD) of NSCLC specimens			
	mIF Batch 1	mIF Batch 2	mIF Batch 3	IHC
		Panel 1		
AE1/AE3	3669.06 ± 776.610	3580.27 ± 1178.88	4502.54 ± 1383.93	3811.73 ± 846.840
PD-L1	3614.95 ± 1986.31	3310.91 ± 2495.13	3531.54 ± 2709.34	0540.51 ± 2127.47
CD4	0891.78 ± 1883.41	976.91 ± 975.64	2218.24 ± 1953.25	633.89 ± 605.81
CD8	539.61 ± 1403.7	107.92 ± 243.08	447.23 ± 339.02	0609.88 ± 1091.16
CD3	0891.78 ± 1883.41	976.91 ± 975.64	2218.24 ± 1953.25	1029.90 ± 1787.23
CD68	0899.55 ± 1374.03	1176.08 ± 836.590	1095.07 ± 683.480	457.70 ± 283.38
		Panel 2		
AE1/AE3	3438.34 ± 1032.16	3496.26 ± 695.430	3769.51 ± 776.440	3811.73 ± 846.840
PD-1	01235.2 ± 1250.32	1239.46 ± 1065.86	1288.12 ± 766.640	170.10 ± 307.71
Granzyme B	55.90 ± 91.55	124.96 ± 80.210	063.68 ± 118.02	398.51 ± 664.76
CD57	1025.41 ± 706.910	637.67 ± 445.85	1054.11 ± 798.790	22.12 ± 48.05
CD45RO	1232.29 ± 1064.19	811.96 ± 513.33	1505.23 ± 1328.76	0797.09 ± 1096.41
FOXP3	0.00 ± 3.32	050.22 ± 118.49	1.49 ± 5.58	186.85 ± 159.02

Note: mIF = multiplex immunofluorescence, IHC = immunohistochemistry.

Although staining variation was observed between mIF batches, overall the markers had similar median value densities (Table 1). The correlation between mIF staining batches exhibited positive and significant correlations as shown in Supplementary Table 2 and Supplementary Figure 3 between batch 1 vs 2 with AE1/AE3, PD-L1, CD4, CD3, CD68, PD-1, CD45RO, and CD57 markers and between batch 1 vs 3 and between batch 2 vs 3 with PD-L1, AE1/AE3, CD4, CD8, CD3, PD-1, CD57, and CD45RO markers. Our image analysis quantification showed that the more consistent marker expressions along the different times of mIF staining were found with AE1/AE3, PD-L1, CD4, CD3, PD-1, CD57, and CD45RO markers followed by CD8, which showed significant and positive correlation in two of three batch comparisons.

**Figure 4:**
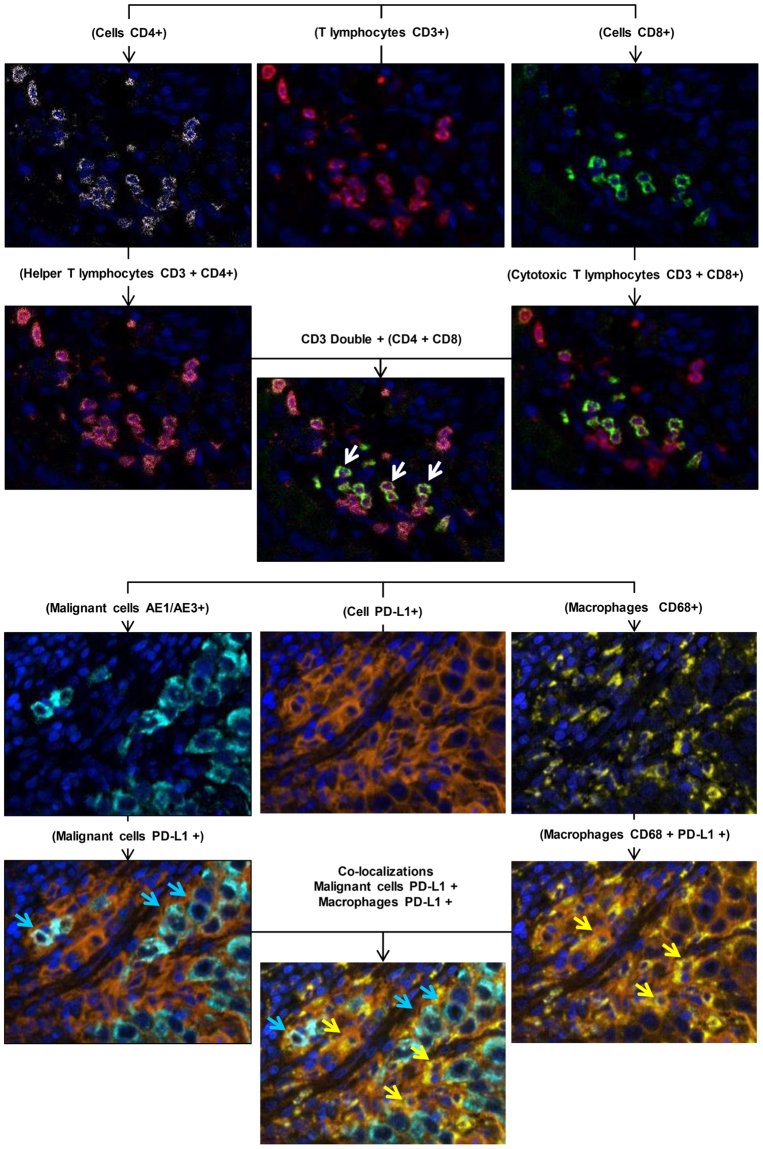
Microphotographs of representative examples of co-localization of the cell markers observed in panel 1.

**Figure 5:**
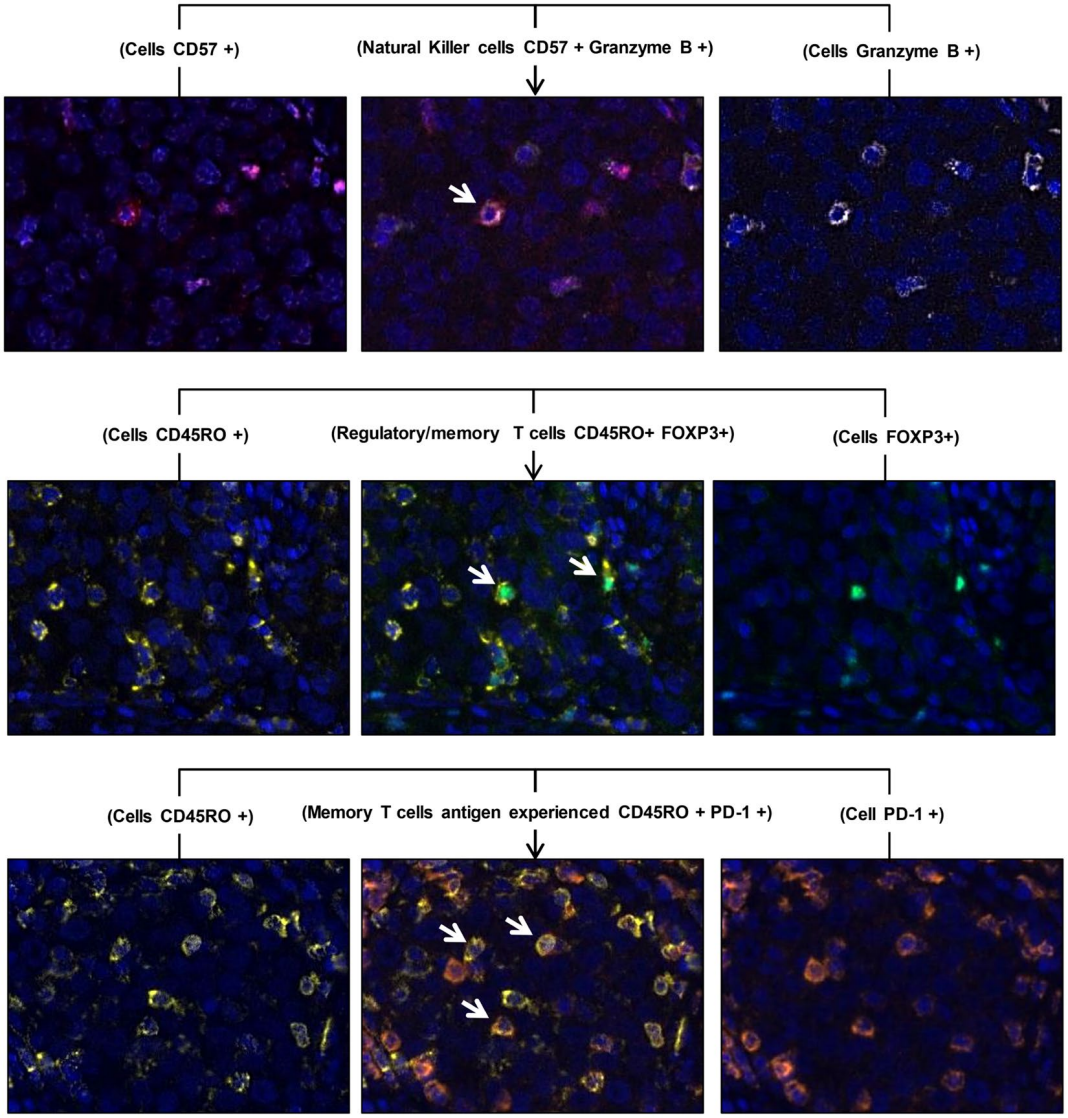
Microphotographs of representative examples of co-localization of the cell markers observed in panel 2.

Studying each individual marker showed that PD-L1–positive membrane expression in malignant cells by chromogenic IHC and mIF (AE1/AE3 plus PD-L1–positive) could be used to identify six positive cases independently of the mIF batch observed, supporting the finding that the PD-L1 (E1L3N) marker used in our study is very efficient for detecting positive PD-L1 tumor cases in a panel of mIF staining. The epithelial marker (AE1/AE3) showed inconsistent staining expression among the batches minimized in these cases by the morphology of the cells and under pathologist supervision during the quantification analysis. Overall, each individual T-cell marker showed staining variation of intensity and density expression among the batches, but the image analysis showed CD68, granzyme B, and FOXP3 to be the more variable staining markers. Cell co-localization was also provided using this mIF panels, showing specific cell phenotypes, including cytotoxic T cells (CD3 plus CD8 positive), helper T cells (CD3 plus CD4 positive), positive PD-L1 epithelial cells (AE1/AE3 plus PD-L1 positive), and positive PD-L1 macrophages (CD68 plus PD-L1 positive) with panel 1 (Fig. 4). Unexpectedly, we observed double CD4/CD8 positive T cells in a small number of CD3 positive cells (Fig. 4). Furthermore, we detected natural killer cells containing cytoplasmic granzyme B (CD57 plus granzyme B positive) but negative CD45RO, regulatory T cells, and memory T cells co-expression (FOXP3 plus CD45RO positive) and PD-1 positive and CD45RO co-expression (PD-1 plus CD45RO positive) with panel 2 (Fig. 5).

## Discussion

In this study, we validated mIF panels using the Opal workflow in the same tissue section to a set of immune marker antibodies, including those against PD-L1 and TAICs, to apply to FFPE tissues. Then we applied those panels in carcinoma tissues to compare and quantify the expression of those markers using mIF and conventional chromogenic IHC. Quantification of mIF marker expression and immune cell population using the Vectra 3.0 multispectral microscopy and image analysis InForm 2.2.1 software (PerkinElmer) showed accurate and reproducible results in the carcinoma cases. The mIF was shown to be an invaluable tool for tumor tissue immune-profiling allowing several targets in the same tissue section and for the development of novel predictive biomarkers for cancer immunotherapy.

The Opal workflow, which allows simultaneous staining of multiple biomarkers within a single paraffin tissue section, was used in the present study. The protocol allows researchers to use antibodies raised in the same species, and each panel was designed specifically for 7-plex IF. The approach involves detection with fluorescent TSA reagents, followed by microwave treatment that removes any nonspecific staining and reduces tissue autofluorescence. After microwave treatment, another round of staining can be performed for additional target detection without risk of antibody cross-reactivity^19^. The Opal kit TSA detection system used in this protocol involves biotinylated secondary antibodies, subsequently labeled with streptavidin enzyme horseradish peroxidase (HRP) and amplified with tyramide-fluorophore conjugates. The TSA detection system is the best for detecting low-expression targets^21,22^, can be performed over 3 days to combined 7 fluorochromes, and can produce reliable results^23,24^. It is important to remember that the concentrations and incubation times of primary antibodies used for IF staining in this study were different from those we used for chromogenic IHC staining.

Overall, we observed that the manual protocol used in our study did not increase or decrease the level of specific signals, and we did not observe nonspecific background with the exception of CD4. Despite the CD4 background, we could identify co-localization of this marker with CD3, and surprisingly we observed small quantities of double-positive CD4+ CD8+ expressing with CD3 in the carcinoma cases, although the secondary antibody was adjusted to eliminate possible nonspecific binding, an important aspect of any multi-antigen staining and a frequently neglected step^23^.

We observed that the approach to the different targets also requires diligent optimization, first in IHC and then in the simplex IF, before mIF staining in control tissues. The use of specific, very well standardized antibodies, as well as the careful use of the other components during staining, is important to obtaining good, reproducible results. AR, performed with use of microwave technology, also requires optimization to ensure both proper AR and endogenous HRP quenching, all the while ensuring complete antibody striping and tissue viability. In addition, properly balanced HRP concentrations are also required to prevent TSA dimer formation, typically achieved through titration of primary antibodies, although this can also be modified through titration of the secondary antibody^19^.

Our experience showed that mIF staining could be performed in paraffin sections from clinical specimens using the same tissue section by several targets. Furthermore, our data suggested that variation in marker expression from the same tissue section can affect the quantification results; for this reason, it is important to use a very well-known control tissue during each staining batch to detect possible errors staining in each panel of mIF. The expression cell variations observed were more deeply remarkable with AE1/AE3, CD68, granzyme B, and FOXP3, suggesting that these markers have less affinity for their epitopes during mIF staining than the other markers have. Although we can discard the idea that the poor reproducibility of these markers in mIF staining is related to the quality of the antibodies used, we cannot discard the possibility that these markers are affected by other unknown conditions during the mIF process and that this problem can be minimized by staining small groups of slides at the same time. Despite this variable cell expression during our staining of mIF carcinoma tissues batches, overall we obtained good reproducibility and convenient staining with successful detection using PD-L1, CD3, CD8, CD57, and PD-1 markers compared with chromogenic IHC staining, and our group successfully multiplexed these biomarkers in two different panels by following our protocol, demonstrating the practical scalability of this method.

Through covalent binding, the TSA-conjugated fluorochromes remained bound to the targeted epitope, allowing for sequential analysis of various targets in mIF staining slides. We observed that TSA, when combined with multispectral image analysis software, such as InForm analysis software, supported this method well, as previously described in the literature^25^. However, this analysis software has limitations since other image analysis software is capable of showing each individual unmixed TSA fluorochrome with a positive signal without noise or aberrant background staining with most of our markers^23^. Compartmental staining (e.g., nuclear, membranous, or cytoplasmic) was also easily obtained for performing the analysis. Co-localization training was allowed in the carcinoma tissues, using the phenotyping tool under pathologist supervision that was essential to analyzing the cells correctly from each panel and detecting or minimizing possible staining errors.

We demonstrated that this method of mIF staining of targets in the same tissue section, if used carefully, is a powerful and efficient tool that allows us to obtain reproducible, reliable, and high-quality staining data. It must be stressed that it is the aim of every immunofluorescence lab to obtain reproducible and accurate results every time, and rigorous steps are necessary to obtain this type of results. There is a plethora of parameters that have significant effects on the outcome of mIF staining. It is common to scrutinize the reagents and protocols used during a mIF staining experiment, for instance reagent concentrations, incubation times, and blocking steps, but in actual fact the results are predisposed from the point of specimen collection onward. First, a careful design of the project is necessary, as is choosing correct reliable antibodies to create a panel of markers to use in the mIF. Second, validation of the antibodies by IHC followed by uniplex IF is also important. Third, with the mIF staining it is important to use fresh tissue sections, regular and thinner cut (maximum 4 µm), in adequately charged slides to avoid poor resolution of tissue morphology and staining. No less important is to identify the appropriate sequence of the targets, and researchers need to determine these through trial and error. Fourth, to diminish manual staining errors and variability of antibody intensity and expression, small quantities of slides can be processed at the same time. Fifth, the use of powerful image analysis software under pathologist supervision is important to detect staining error and determine the correct analysis.

The implementation of mIF in the same tissue section and the flexibility to create panels to different targets, offers many opportunities for innovative digital image analysis approaches (inflammatory tumor infiltration, cell phenotyping, proximity, 3D-reconsatruction), increasing the novelty of this methodology. Additionally, application of this type of methodology to answer scientific research question or testing hypothesis of clinical importance could provide answers to different questions. Furthermore, localization of multiple targets in the same tissue section will provide unique insight into spatial, cell type, and even phenotype co-localization–type specific distribution of molecules of interest. It allows deeper understanding of the tumor microenvironment during development and can establish conditions before and after treatment.

## Electronic supplementary material

Supplementary Figures and Tables
