# Supplementary material for: Validation of multiplex immunofluorescence panels using multispectral microscopy for immune-profiling of formalin-fixed and paraffin-embedded human tumor tissues
Source: Sci Rep. 2017 Oct 17;7:13380. doi: 10.1038/s41598-017-13942-8 (PMC5645415; doi:10.1038/s41598-017-13942-8)

Validation of multiplex immunofluorescence panels using multispectral microscopy for immune-profiling of formalin-fixed and paraffin-embedded human tumor tissues

Edwin R. Parra, Naohiro Uraoka, Mei Jiang, Pamela Cook, Don Gibbons, Marie-Andrée Forget, Chantale Bernatchez, Cara Haymaker, Ignacio I. Wistuba and Jaime Rodriguez-Canales.

Supplementary Table 1. Correlation between chromogenic immunohistochemistry (IHC) and multiplex immunofluorescence (mIF) groups.

Marker	*mIF Batch 1 vs IHC	*mIF Batch 2 vs IHC	*mIF Batch 3 vs IHC
		**Panel 1**	
AE1/AE3	r = 0.273<br>*P* = 0.417	r = 0.527<br>*P* = 0.096	r = 0.200<br>*P* = 0.555
PD-L1	r = 0.618<br>*P* = 0.043	r = 0.691<br>*P* = 0.019	r = 0.627<br>*P* = 0.039
CD4	r = 0.709<br>*P* = 0.015	r = 0.764<br>*P* = 0.006	r = 0.855<br>*P* = 0.001
CD8	r = 0.764<br>*P* = 0.006	r = 0.555<br>*P* = 0.077	r = 0.773<br>*P* = 0.005
CD3	r = 0.791<br>*P* = 0.004	r = 0.782<br>*P* = 0.005	r = 0.909<br>*P* = 0.0001
CD68	r = 0.173<br>*P* = 0.612	r = 0.382<br>*P* = 0.247	r = 0.873<br>*P* = 0.001
		**Panel 2**	
AE1/AE3	r = 0.427<br>*P* = 0.190	r = 0.655<br>*P* = 0.029	r = 0.382<br>*P* = 0.247
Granzyme B	r = 0.173<br>*P* = 0.612	r = 0.018<br>*P* = 0.958	r = 0.400<br>*P* = 0.223
CD57	r = 0.764<br>*P* = 0.006	r = 0.527<br>*P* = 0.096	r = 0.746<br>*P* = 0.009
CD45RO	r = 0.300<br>*P* = 0.370	r = 0.346<br>*P* = 0.298	r = 0.246<br>*P* = 0.467
PD-1	r = 0.846<br>*P* = 0.001	r = 0.918<br>*P* < 0.0001	r = 0.836<br>*P* = 0.001
FOXP3	r = 0.434<br>*P* = 0.183	r = 0.410<br>*P* = 0.210	r = 0.229<br>*P* = 0.499

Note: *Spearman correlation.

Supplementary Table 2. Correlation between multiplex immunofluorescence staining batches.

Marker	*Batch 1 vs Batch 2	*Batch 1 vs Batch 3	*Batch 2 vs Batch 3
	**Panel 1**		
AE1/AE3	r = 0.836<br>*P* = 0.001	r = 0.800<br>*P* = 0.003	r = 0.864<br>*P* = 0.001
PD-L1	r = 0.773<br>*P* = 0.005	r = 0.718<br>*P* = 0.013	r = 0.800<br>*P* = 0.003
CD4	r = 0.918<br>*P* < 0.0001	r = 0.827<br>*P* = 0.002	r = 0.746<br>*P* = 0.009
CD8	r = 0.200<br>*P* = 0.555	r = 0.709<br>*P* = 0.015	r = 0.627<br>*P* = 0.039
CD3	r = 0.918<br>*P* = 0.0001	r = 0.827<br>*P* = 0.002	r = 0.746<br>*P* = 0.009
CD68	r = 0.700<br>*P* = 0.017	r = 0.318<br>*P* = 0.340	r = 0.336<br>*P* = 0.312
	**Panel 2**		
AE1/AE3	r = 0.891<br>*P* = 0.0002	r = 0.655<br>*P* = 0.029	r = 0.600<br>*P* = 0.051
CD57	r = 0.736<br>*P* = 0.010	r = 0.927<br>*P* < 0.0001	r = 0.836<br>*P* = 0.001
Granzyme B	r = 0.482<br>*P* = 0.133	r = 0.482<br>*P* = 0.133	r = 0.555<br>*P* = 0.077
CD45RO	r = 0.727<br>*P* = 0.011	r = 0.882<br>*P* = 0.0003	r = 0.755<br>*P* = 0.007
PD-1	r = 0.918<br>*P* < 0.0001	r = 0.936<br>*P* < 0.0001	r = 0.927<br>*P* < 0.0001
FOXP3	r = 0.133<br>*P* = 0.696	r = 0.575<br>*P* = 0.064	r = 0.430<br>*P* = 0.187

Note: *Spearman correlation.

Supplementary Figure 1. Microphotographs of representative examples of validation from chromogenic IHC and uniplex IF PD-L1 expression. PD-L1 shown membranous expression in (a) epithelial tonsil crypts, (b) placenta syncytiotrophoblasts, and (c) Hodgkin disease–derived cell line (HDLM2), and negative PD-L1 expression in (d) prostate cancer cell line (PC3). ×200 magnification.

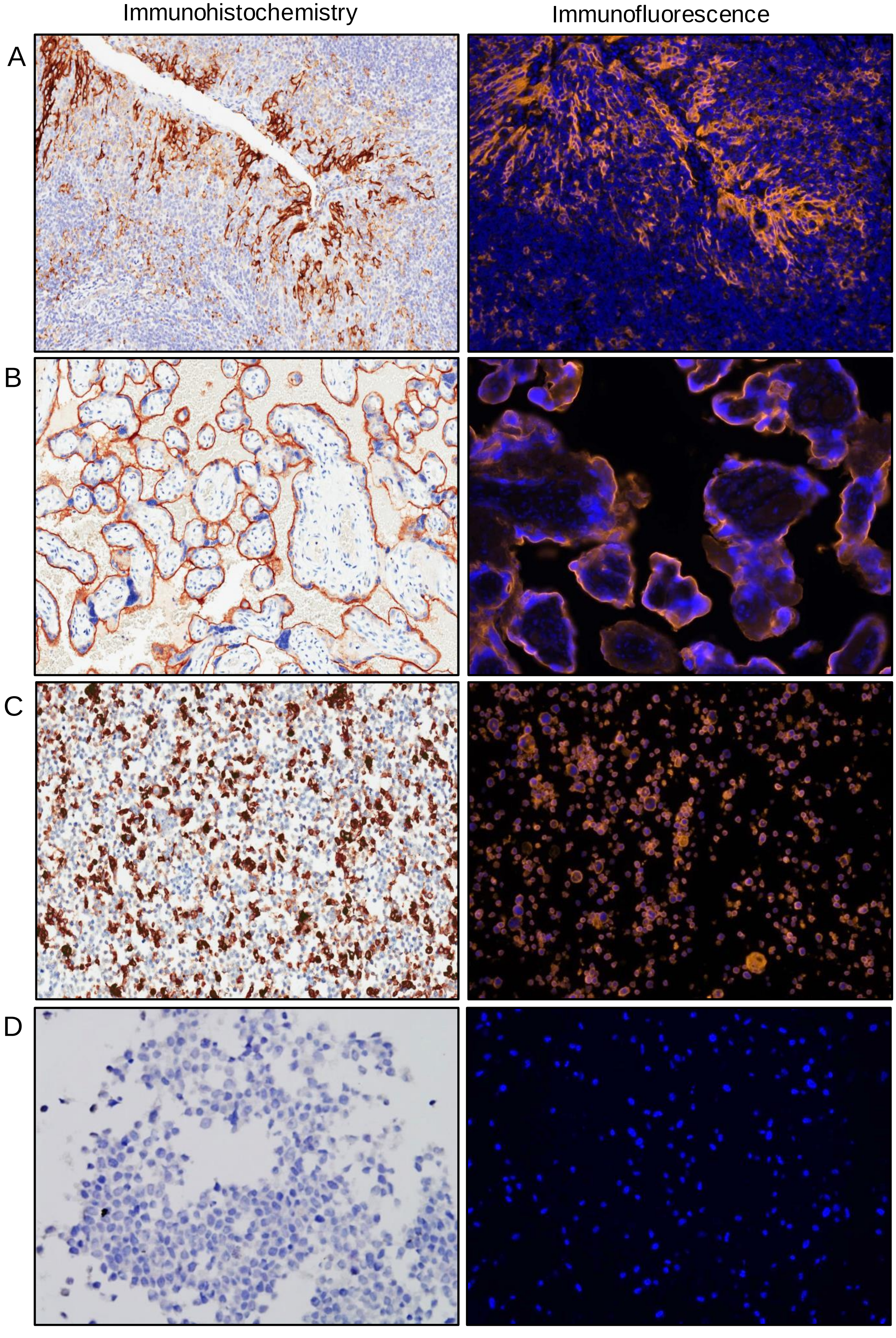

Supplementary Figure 2. Microphotographs of representative examples of staining variation between staining times in panels 1 and 2. Multiplex IF markers in a case of non–small cell lung carcinoma. ×200 magnification.

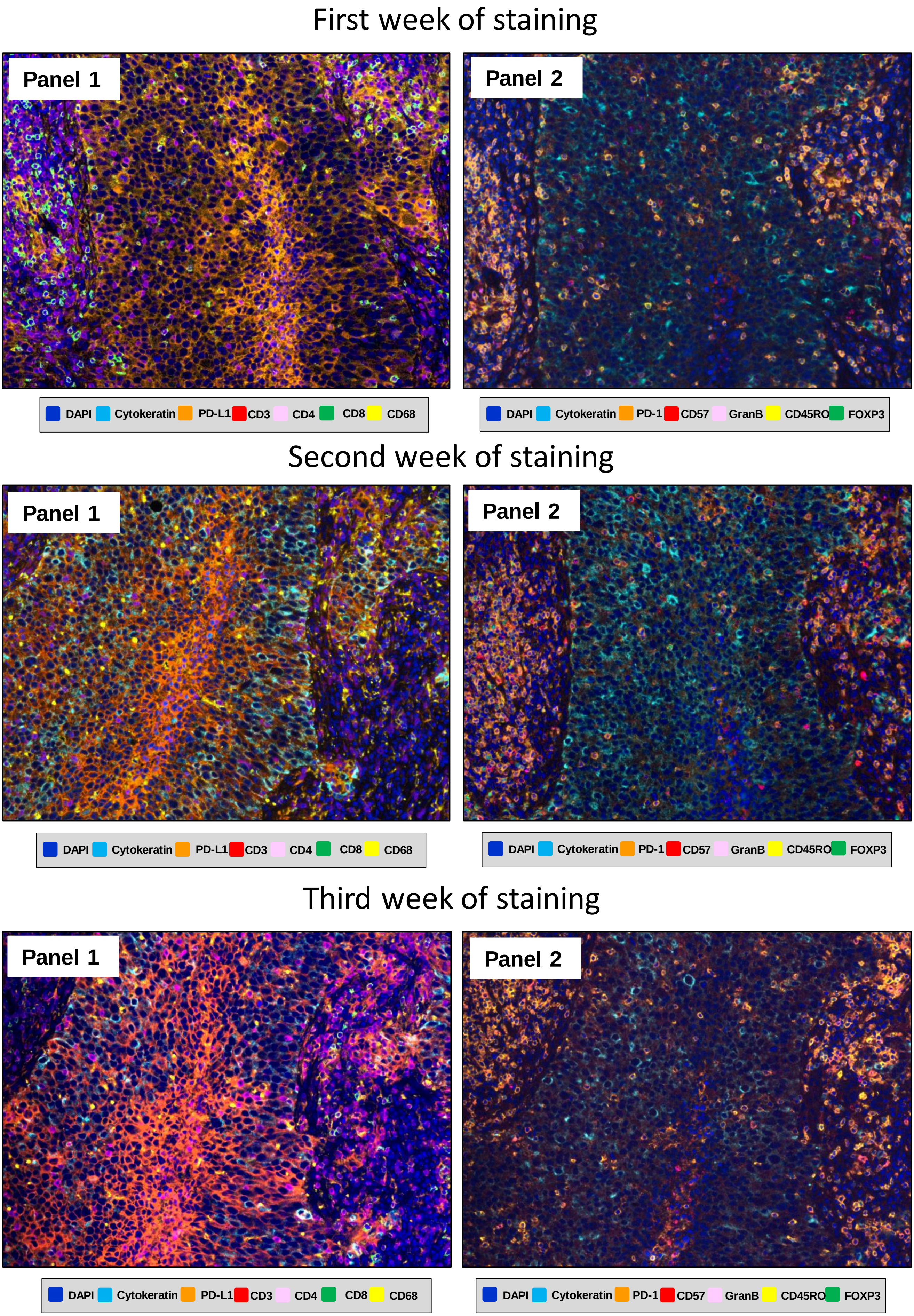

Supplementary Figure 3. Scatter plots compare the correlation between IHC densities and multiplex IF markers in panels 1 and 2 in the different batches.

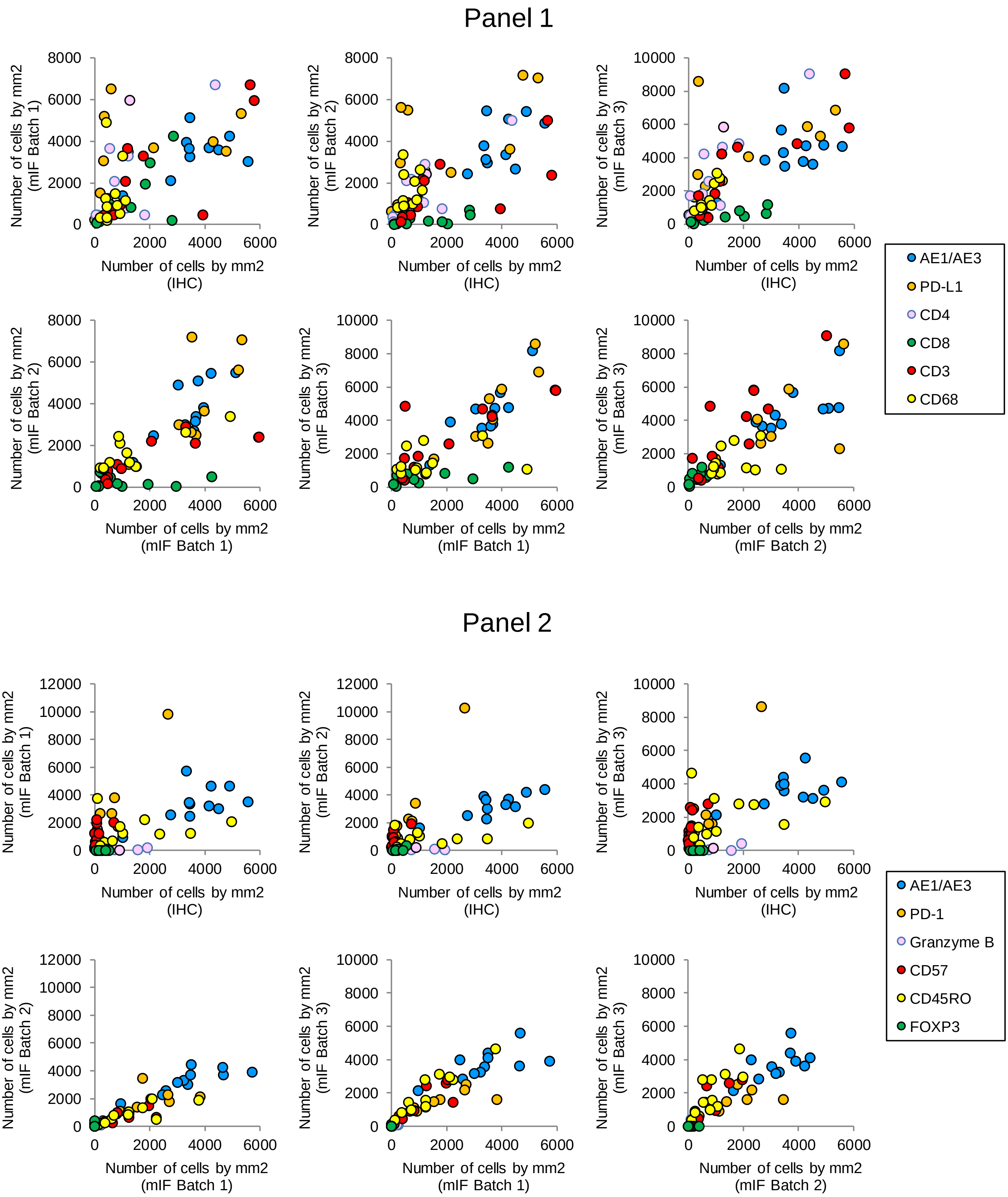

Supplement: Supplementary file 1 — Supplementary Figures and Tables [file 41598_2017_13942_MOESM1_ESM.pdf]
